# A Combined Approach of NMR and Mass Spectrometry Techniques Applied to the α-Cyclodextrin/Moringin Complex for a Novel Bioactive Formulation †

**DOI:** 10.3390/molecules23071714

**Published:** 2018-07-13

**Authors:** David Mathiron, Renato Iori, Serge Pilard, Thangavelu Soundara Rajan, David Landy, Emanuela Mazzon, Patrick Rollin, Florence Djedaïni-Pilard

**Affiliations:** 1 Plateforme Analytique, Institut de Chimie de Picardie FR 3085 CNRS, Université de Picardie Jules Verne, 33 rue St Leu, 80039 Amiens, France; serge.pilard@u-picardie.fr; 2 Consiglio per la Ricerca in Agricoltura e L’analisi Dell’economia Agraria, Centro di Ricerca Agricoltura e Ambiente (CREA-AA), Via di Corticella 133, 40128 Bologna, Italy; renato.iori48@gmail.com; 3 Istituti di Ricovero e Cura a Carattere Scientifico, IRCCS Centro Neurolesi “Bonino-Pulejo”, Via Provinciale Palermo, Contrada Casazza, 98124 Messina, Italy; tsrajanpillai@gmail.com (T.S.R.); emazzon.irccs@gmail.com (E.M.); 4 Unité de Chimie Environnementale et Interactions sur le Vivant (UCEIV, EA 4492), ULCO, F-59140 Dunkerque, France; david.landy@univ-littoral.fr; 5 Institut de Chimie Organique et Analytique (ICOA), Université d’Orléans et CNRS, UMR 7311, BP 6759, F-45067 Orléans, France; patrick.rollin@univ-orleans.fr; 6 Laboratoire de Glycochimie, des Antimicrobiens et des Agroressources UMR 7378, Université de Picardie Jules Verne, 33 rue St Leu, 80039 Amiens, France

**Keywords:** *Moringa oleifera*, cyclodextrin, moringin, isothiocyanate, anti-inflammatory, nuclear magnetic resonance (NMR), mass spectrometry (MS)

## Abstract

Moringin, obtained via enzymatic conversion of the glucosinolate precursor glucomoringin, is an uncommon member of the isothiocyanate class, and has been proven to possess a broad range of biological activities such as antitumor activity, protection against neurodegenerative disorders and bactericidal effects. Since moringin is weakly soluble in water and unstable in aqueous medium, cyclodextrins (CDs) were considered for the development of a new moringin formulation, with a view to improving its solubility and stability in aqueous solution for use as an anti-inflammatory. A combined structural study using proton nuclear magnetic resonance (^1^H-NMR), diffusion-ordered spectroscopy (DOSY) and ion mobility mass spectrometry (IM-MS) is reported, highlighting the formation of a 1:1 α-CD/moringin inclusion complex. The association constant *K* was determined (1300 M^−1^ at 300 K). Completion of the structural characterization was performed by T-ROESY and MS/MS experiments, which evidenced the mode of penetration of moringin into α-CD. Finally, the “chaperone-like” properties of α-CD with respect to the stability of moringin have been highlighted.

## 1. Introduction

**Figure 1:**
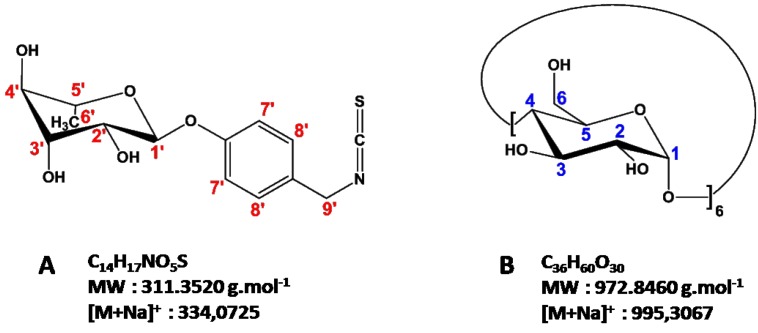
Structures of (**A**) moringin (MOR) and (**B**) α-cyclodextrin (α-CD).

In the botanical order Brassicales, isothiocyanates (ITCs) are a well-established group of naturally-occurring products, which are not produced as such by the plant, but rather released after cell damage by the enzymatic action of myrosinase (β-thioglucoside glucohydrolase; E.C. 3.2.1.147) on their glucosinolate (GL) precursors [1]. To date more than 130 GLs have been identified in plants [2]. Glucomoringin (GMG) is an atypical member of the GL family present in tropical vegetables belonging to the family Moringaceae—namely *Moringa oleifera* Lam, commonly known as the “horseradish tree”. This most widely cultivated species produces seeds which currently contain 8–10% of GMG. The chemical structure of GMG (4-(α-l-rhamnopyranosyloxy)benzyl GL) is quite unusual as it represents a *O*-glycosylated form of glucosinalbin, a phenolic GL widespread in several families of the order Brassicales. Controlled myrosinase hydrolysis in phosphate buffer (PBS) of GMG readily releases the corresponding isothiocyanate 4-(α-l-rhamnopyranosyloxy)benzyl ITC (moringin, MOR) as displayed in Figure 1A:

Moringin was recently characterized [3,4] and proven to display a broad range of biological activities, including protective effects against neurodegenerative disorders [5,6,7], effective antitumor promoting activity [8], apoptosis induction in in vitro and in vivo models such as myeloma [9], astrocytoma [10] and antimicrobial effects [11,12]. In previous studies, we investigated the neuroprotective and antitumor efficacy of a PBS solution of MOR obtained from GMG by myrosinase-catalyzed hydrolysis instead of using the isolated pure ITC [13]. MOR is a solid, odourless compound, which is stable at room temperature and can easily be prepared in large amounts from GMG. It is thus markedly different from other natural bioactive ITCs—e.g., sulforaphane—which are volatile liquids, with pungent smells.

Nevertheless, ITCs, including MOR, are in general poorly soluble [14] and degrade slowly in water [15], which results in stability problems for the delivery in an enriched form. This difficulty can be overcome by complexation of ITCs with cyclodextrins (CDs) [15,16]. CDs are cyclic oligosaccharides derived from starch consisting of 6, 7 and 8 α-1,4-linked d-glucopyranoside units and for the most common called α-, β- and γ-CD, respectively. Their truncated cone-like three-dimensional structure endows them with inclusion properties and because of the large number of hydroxyl groups oriented toward the outside of the cone, CDs are water soluble, in particular α-CD (160 mM at 25 °C) (Figure 1B). These biocompatible, cyclic oligosaccharides are able to form with small molecules water-soluble inclusion complexes that are useful for pharmaceutical applications since they do not elicit immune responses and have low toxicities in animals and humans [17]. Recently, we investigated the anti-inflammatory effects of a new formulation of MOR containing α-CD, using in vitro lipopolysaccharide (LPS)-stimulated RAW264.7 macrophage cells, a commonly used model for inflammation studies [18]. The (MOR/α-CD) mixture showed anti-inflammatory effects in LPS activated macrophages, suggesting a promising therapeutic approach for inflammatory diseases. The aim of the present work is (a) to estimate the stability and bioavailability in aqueous media of various formulations of MOR with and without α-CD by studying their cytoprotective properties and (b) to investigate the interactions between MOR and α-CD in aqueous medium by physico-chemical studies combining NMR and mass spectrometry experiments to better understand the mechanism involved.

## 2. Results and Discussion

### 2.1. Investigation of Anti-Inflammatory Properties 

GMG was isolated from *M. oleifera* L. seeds according to previously reported methods [9,19]. Pioneering inclusion experiments of MOR, produced in situ by myrosinase hydrolysis of GMG, into α-cyclodextrin were initially performed by Roselli et al. [20]. It was found that α-CD was effective in both solubilizing and stabilizing MOR. With regard to its biological activity, we have demonstrated in our previous preclinical studies that MOR exerts exemplary anti-inflammatory effects against neurodegenerative diseases [21,22]. Recently, we have shown the anti-inflammatory effect of the α-CD/MOR complex in LPS-stimulated macrophages [18].

**Figure 2:**
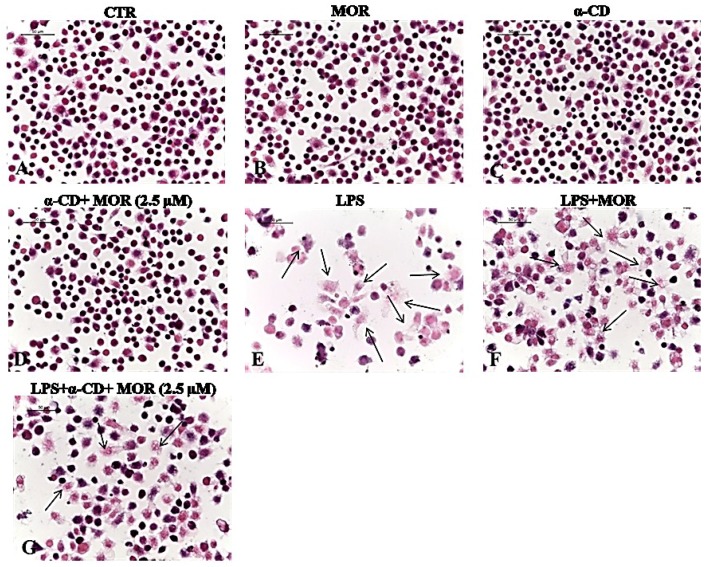
Cytoprotective effect of α-CD/MOR in LPS-stimulated RAW macrophages. Eosin and haematoxylin stain (E&H) staining displayed no morphological changes or cell loss in untreated control (**A**); MOR alone (**B**); α-CD alone (**C**) and α-CD/MOR (2.5 μM) (**D**) treated macrophages. Increased cell size (black arrows) and cell death was observed in LPS-stimulated macrophages (**E**). LPS-stimulated elongated macrophages were less with relative increase in cell numbers in one week old α-CD+MOR-pretreated macrophages (**G**) than that of macrophages pretreated with one week old MOR alone (**F**). All images were taken at 40× (bar 50 µm).

In the present study, we have investigated the stability of the same complex with reference to its biological properties and compared it with MOR alone. To this end, we have studied the cytoprotective properties of MOR either alone or in the presence of α-CD, the compounds being used either as fresh solutions or one-week old solutions, in LPS-stimulated RAW macrophages. Eosin and hematoxylin (E & H) staining showed that macrophages treated with MOR alone, α-CD alone and α-CD/MOR mixture did not induce toxic effects (Figure 2B–D, respectively) and normal cell sizes were compared with untreated control cells (Figure 2A). As shown in Figure 2E, macrophages showed increased cell size followed by a reduction in overall cell population due to the cytotoxic inflammatory action elicited by LPS. Fresh solutions of both MOR and α-CD/MOR complex display cytoprotective effects in LPS-induced macrophages (data not shown). Conversely, we also found that the cytoprotective effect of one-week old MOR solutions was considerably reduced when compared with one-week old α-CD/MOR complex solution. LPS-activated elongated macrophages were less with relative increase in cell numbers in one-week old α-CD/MOR-pretreated macrophages (Figure 2G) than that of macrophages pretreated with one-week old MOR alone (Figure 2F). Those results demonstrate that the α-CD/MOR complex has extended biostability compared to MOR alone, suggesting an enhanced therapeutic potential of MOR through complexation with α-CD.

### 2.2. Physico-Chemical Studies of α-CD/MOR Mixture 

Only few examples of inclusion complexes between glucosinolates or ITCs and CDs are reported in literature. Ohta et al. reported a decrease in decomposition of allyl ITC in aqueous media in the presence of CDs [15]. In other respects, interactions between butyl ITC and α-CD were highlighted by proton nuclear magnetic resonance (^1^H-NMR) experiments in aqueous solutions and X-ray crystallography [14]. Moreover, the same authors described the inclusion complex between α-CD and glucotropaeolin, the glucosinolate precursor of benzyl ITC [23]. The action of myrosinase on this inclusion complex was shown to lead directly to the corresponding benzyl-ITC/α-CD complex without dissociation. More recently W. Li et al. claimed the preparation and characterization of benzyl-ITC/β-CD complex [16]. It should be pointed out that the NMR experiments were performed in DMSO, a well-known dissociating solvent. Nevertheless, in most cases, the described inclusion complexes between CDs and ITCs were not characterized unambiguously in terms of stoichiometry, association constant and three-dimensional structure in aqueous media.

#### 2.2.1. Evidence on the α-Cyclodextrin/Moringin (α-CD/MOR) Interactions

**Figure 3:**
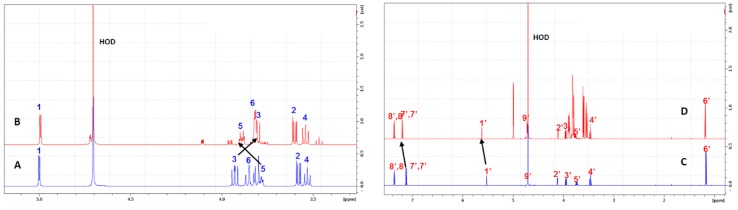
Partial proton nuclear magnetic resonance (^1^H-NMR) spectra (5 mM in D_2_O) of α-CD in the absence (**A**) and in the presence of MOR (**B**) recorded at 600 MHz at 300 K and of MOR in the absence (**C**) or in presence of α-CD (**D**).

To confirm the formation of inclusion complexes between α-CD and MOR, ^1^H-NMR experiments were first carried out (Figure 3). A comparison of ^1^H-NMR spectra of α-CD (5 mM in D_2_O) in the presence and absence of MOR was performed to highlight chemical shift variations on the H3 and H5 protons located inside the α-CD cavity. The addition of MOR to CD led to upfield and downfield chemical shift variations on H3 and H5 respectively, consistent with the formation of an inclusion complex. Due to the so-called anisotropic effect, the difference of variation of the chemical shift observed between H3 and H5 indicates that both protons may be placed in different environments. In other words, we can assume that each proton, located inside the α-CD cavity exhibits specific position with respect to MOR. In the case of MOR, it should be noted that the largest chemical shift variations were observed in the presence of α-CD for H7’, H8’ and H9’, located on the ITC moiety.

**Figure 4:**
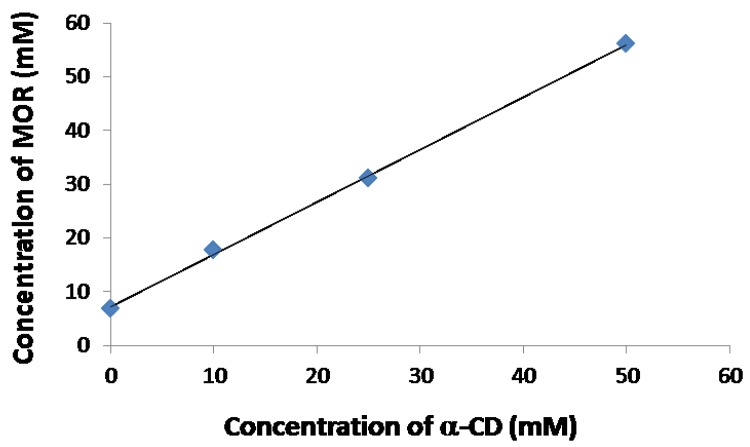
Phase solubility diagram of MOR in D_2_O solution in presence of α-CD obtained by ^1^H-NMR (600 MHz, 300 K).

To probe the potential solubilizing effect of α-CD on MOR in water, a phase solubility diagram was performed according to the Higuchi and Connors method using a quantitative ^1^H-NMR experiment. As depicted in Figure 4, a linear increase in MOR solubility in D_2_O as a function of α-CD concentration was observed revealing an A_L_ type profile and confirming the formation of a α-CD/MOR complex in solution. Moreover, it should be pointed out that MOR undergoes a dramatic improvement of its solubility in the presence of α-CD since its concentration at saturation in D_2_O goes from 6.9 mM in the absence of α-CD to 56.1 mM with 50 mM of α-CD.

**Figure 5:**
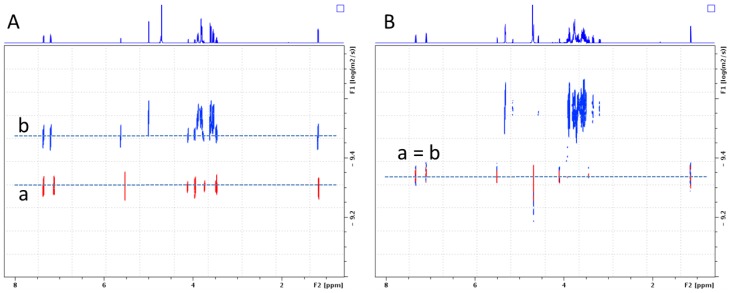
Superimposed NMR diffusion-ordered spectroscopy (DOSY) spectra recorded at 600 MHz (D_2_O, 300 K, 5 mM) of MOR (**A**) alone (red) and in the presence of α-CD (blue); (**B**) alone (red) and in the presence of maltohexaose (blue). Vertical scale: diffusion coefficient; horizontal scale: ^1^H chemical shift.

To gain additional evidence on the α-CD/MOR interaction and on the formation of an inclusion complex, diffusion experiments in liquid phase using proton NMR with diffusion-ordered spectroscopy (^1^H DOSY NMR) and in gas phase with ion mobility separation coupled to mass spectrometry (IMS-MS) were considered. First, ^1^H DOSY NMR experiments also confirmed the interaction of MOR with α-CD due to variations in the diffusion coefficient D value as displayed in Figure 5A. The diffusion coefficient can be linked to the translational motion of molecules in solution and its value depends on the size of the object [24]. The bigger the molecule is, the smaller the D value will be. For instance, MOR alone in D_2_O revealed a D value of 5.01 × 10^−10^ m^2^/s (Figure 5A,a) whereas a value of 3.17 × 10^−10^ m^2^/s was observed for α-CD alone. In the presence of α-CD, a decrease in the MOR D value was observed, reaching a value of 3.38 × 10^−10^ m^2^/s (Figure 5A,b) very close to that of α-CD alone, revealing an interaction that can be attributed to the formation of a α-CD/MOR inclusion complex. The same approach was envisaged with a linear oligosaccharide maltohexaose (MH) and revealed that no interaction between MH and MOR occurred due to the absence of D_MOR_ value change (Figure 5B, a = b). Since the main difference between α-CD and MH is their 3D structure (truncated cone versus linear structure), this observation seems to indicate specific interactions between α-CD and MOR in solution.

To confirm this result, mass spectrometry, widely used to highlight non-covalent interaction in the gas phase under native conditions, was considered [25].

**Figure 6:**
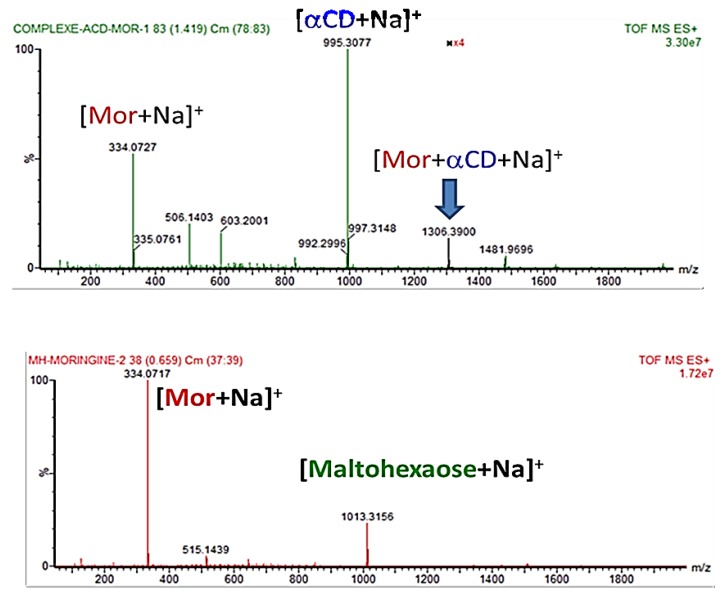
Time-of-flight mass spectrometry (TOF MS) spectra of equimolar mixtures of MH/MOR (**A**) and of α-CD/MOR (**B**) in water (0.5 mM) recorded in positive electrospray ionization mode (ESI^+^).

To do this, equimolar mixtures of MH/MOR and α-CD/MOR were infused in the electrospray source operating in positive ionization mode (ESI^+^) to give mass spectra depicted in Figure 6. For MH/MOR mixture (Figure 6A), sodium adducts for MOR and MH were observed respectively at *m*/*z* 334.07 and 1013.32. However, no *m*/*z* corresponding to the association of MOR and MH was detected revealing the absence of MH/MOR interaction in gas phase as previously shown in liquid phase by DOSY NMR. For α-CD/MOR mixture additional ions were observed (Figure 6B), in particular a sodium adduct corresponding to the association of one α-CD to one MOR at *m*/*z* 1306.39 suggesting an interaction with 1:1 stoichiometry. As expected, since we are in presence of a non-covalent complex which is in equilibrium with the dissociated forms of both partners, sodium adducts of MOR and α-CD were observed at *m*/*z* 334.07 and 995.31, respectively.

Furthermore, the absence of non-specific association between MH and MOR and the presence of a *m*/*z* corresponding to the mass of α-CD/MOR complex corroborate that α-CD and MOR interacts by an inclusion process and not simply by the formation of an electrostatic adduct involving the outside of α-CD [26].

**Figure 7:**
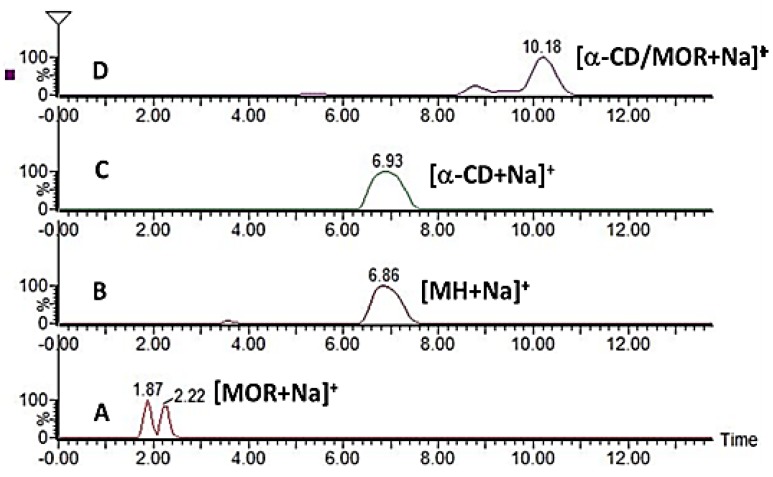
Comparison of reconstructed mobility traces of sodium adduct ions of MOR (**A**), MH (**B**), α-CD (**C**) and an equimolar mixture of α-CD/MOR (**D**) with following ion mobility separation (IMS) parameters: wave velocity (WV) = 600 m/s, wave height (WH) = 40 V.

In order to gain insights into the conformational dynamics of a system, diffusion experiments in the gas phase involving ion mobility separation hyphenated with mass spectrometry (IM-MS) has been of growing interest over the last few years [27]. Ion mobility introduces an additional dimension of separation to mass spectrometry, allowing the characterization and comparison of dynamic changes in analyte structures, in particular when specific noncovalent associations such as CD/guest complex occur [28]. Indeed, the ion drift time (t_d_) in the mobility cell is directly proportional to the three-dimensional ion shape. Thus, if an interaction in the gas phase between CD and another molecule exists, the CD original drift time will be shifted to a higher value, evidencing the formation of a supramolecular assembly. Mobilograms of selected [M + Na]^+^ ions corresponding to MOR, MH and α-CD alone as well as α-CD/MOR mixture are displayed in Figure 7. It should be pointed out that the drift times of MH (B) and α-CD (C) are similar due to close chemical structures. In contrast in the presence of MOR, the α-CD drift time is shifted (D) indicating a modification of its size and shape. This result is in agreement with MOR inclusion in the α-CD cavity, which is not the case for MH/MOR mixture where no drift time shift was observed.

#### 2.2.2. Characterization of the α-CD/MOR Inclusion Complex

**Figure 8:**
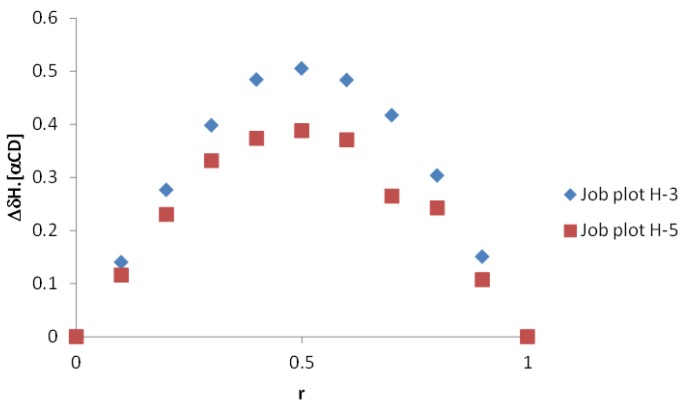
Continuous variation plot (Job Plot) for H3 and H5 protons of α-CD (600 MHZ, D_2_O, 300 K).

The MS observations in the gas phase are in complete accordance with NMR diffusion experiments highlighting the complementarity of our combined MS-NMR approach. Preliminary mass spectrometry data enabled direct access to the stoichiometry of α-CD/MOR complex by analyzing the observed masses on the spectrum (Figure 6) suggesting a 1:1 interaction. This approach can be used for the rapid screening of different complexes but remains uncertain due to the formation of multimer clusters of CDs during the ESI process. So, to unambiguously determine α-CD/MOR complex stoichiometry, NMR was used. In the case of fast exchange rate, frequently encountered with cyclodextrins, the stoichiometry of the complex could not be directly determined, and the continuous variation’s method so-called “Job’s method” based on ^1^H-NMR titration experiments was used [29]. This method required that the total concentration of α-CD and MOR was kept constant (10 mM) with the ratio *r* being varied from zero to one. Plots of the observed *Δδ*·[α-CD] as a function of *r* led to Job plots (Figure 8). In all cases, the Job plots showed a maximum at *r* = 0.5 and a symmetrical shape in agreement with the complex has 1:1 stoichiometry for the complex confirming the observed result in MS.

**Figure 9:**
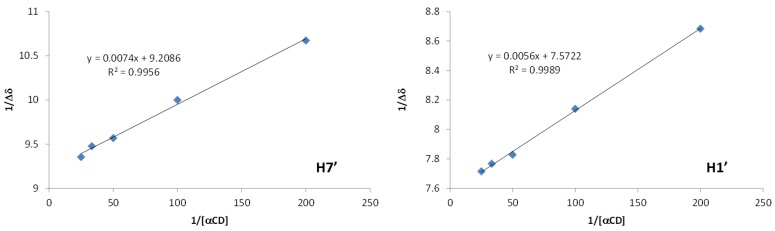
Benesi–Hildebrand Plots for protons of MOR (600 MHz, D_2_O, 300 K, 0.2 mM) in the presence of α-CD.

The apparent association constant *K* was determined by the Benesi–Hildebrand method [29]. The MOR concentration was set to 0.2 mM and that of the α-CD varied between 5 and 40 mM at 300 K (Figure 9). The average association constant *K* was evaluated from several protons (H1’ and H7’) of the MOR to yield a value of 1300 M^−1^ at 300 K. This *K* value must be compared with those obtained for few complexes described in the literature: 390 M^−1^ for allyl-ITC/α-CD [16], 36 M^−1^ for allyl-ITC/β-CD [30] or 600 M^−1^ for benzyl-ITC/α-CD [16]. α-CD seems to be the most relevant cyclodextrin for the inclusion complex formation with ITC derivatives and the presence of an aromatic part in the guest molecule appears to stabilize the complex in solution.

**Figure 10:**
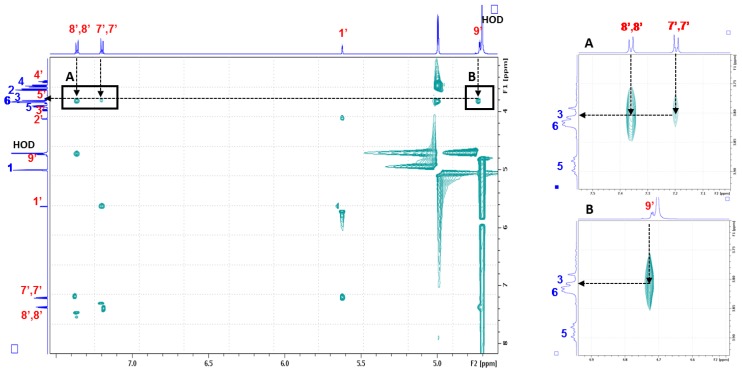
Partial contour plot of transverse rotating-frame Overhauser enhancement spectroscopy (T-ROESY) experiment (spin lock field strength 1250 Hz, 600 MHz, D_2_O, 300 K) performed on 5 mM equimolar α-CD/MOR mixture with expansion (**A**,**B**) of intermolecular cross-peaks between MOR and α-CD.

Finally, we focused on the characterization of the α-CD/MOR inclusion complex using transverse rotating-frame Overhauser enhancement spectroscopy (T-ROESY) experiments. The implementation of NMR experiments based on the study of dipole interactions such as the ROESY pulse sequence makes it possible to highlight the protons involved in the complexation process. But, they can also show correlation signals from a transfer of type total correlation spectroscopy-ROESY (TOCSY-ROESY) or ROESY-TOCSY that can disturb the interpretation. This phenomenon is particularly favored in the case of cyclodextrins this is why a T-ROESY experiment is routinely used for the characterization of cyclodextrin complexes in solution [31] As displayed in Figure 10, the presence of cross-correlation peaks between specific protons of MOR and those from the α-CD cavity fully supports the formation of an inclusion complex. Strong interactions between the H3 protons of α-CD and protons of the ITC moiety (mainly H8’,8’ and H9’ and also weakly H7’,7’) were observed. It should be noted that no interaction with H5 and H6 of α-CD was observed and that the rhamnopyranosyl moiety did not seem to be involved on the inclusion process.

**Figure 11:**
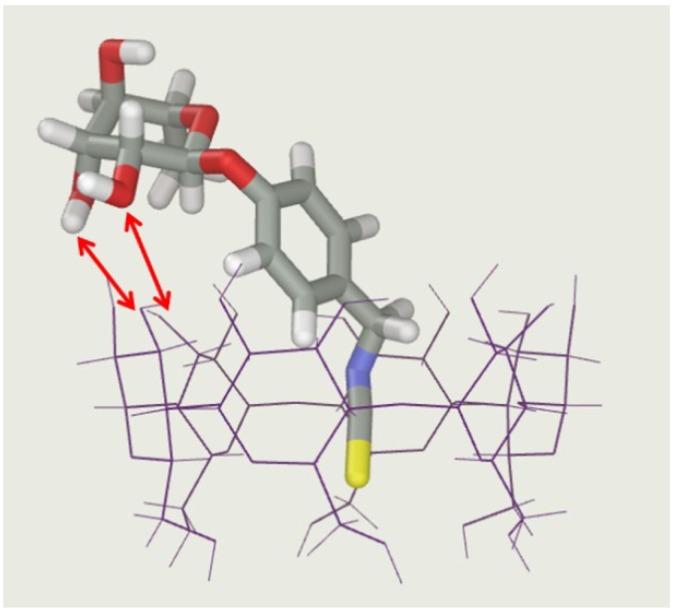
Example of α-CD/MOR conformation extracted from molecular dynamics simulation. Red arrows underline possible hydrogen bonds between MOR and α-CD.

Molecular dynamics simulations (MD) have been performed on the basis of this structural hypothesis, in order to illustrate the steric feasibility of such an inclusion mode. The mean values obtained for the complexation energy (−22.1 kcal/mol) and intermolecular energy (−19.1 kcal/mol) strongly support the favorable character of this inclusion, at least from an enthalpic point of view. The different structures observed during the MD trajectories confirm the significant complementarity between the two partners, with a deep insertion of the isothiocyanate group. As exemplified by one of these conformations displayed in Figure 11. The binding of MOR to α-CD occurs via the inclusion of *p*-hydroxybenzyl ITC moiety into the cavity of α-CD leaving the Rhamnose residue of MOR in an external position, suitable for interactions with secondary hydroxyls of α-CD via temporary hydrogen bonds. The mean distance between the protons of MOR and the two nearest H3 protons of α-CD is equal to 2.5 for H-9’, 3.1 Å. for H-8’ and 4.0 for H,7’, which is in fair agreement with ROESY data.

**Figure 12:**
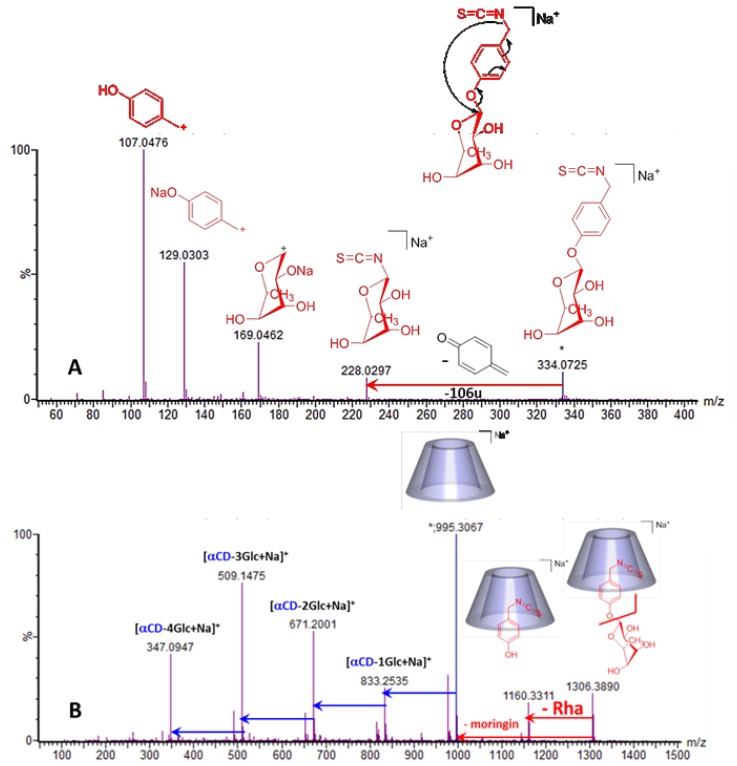
Mass spectrometry (MS/MS) spectra of water solutions of MOR alone (0.5 mM) (**A**) and in presence of α-CD (0.5 mM) (**B**) recorded respectively at a collision energy of 25 eV and 95 eV.

To gain deeper structural information on the α-CD/MOR inclusion complex, MS/MS was first performed on MOR alone (*m*/*z* 334.07) to obtain a reference mass spectrum and then on α-CD/MOR inclusion complex (*m*/*z* 1306.39) to evaluate the influence of α-CD on the MOR fragmentation pathway and on a possible stabilizing effect. On the MS/MS spectrum of MOR performed at 25 eV (Figure 12A), we observed that the main fragment ions at *m*/*z* 107.05 and 129.03 can be assigned to *p*-cresol structure. The ion at *m*/*z* 228.03 was obtained by a loss of the *p*-hydroxybenzyl part (−106u) from MOR due to a 6-membered transition state rearrangement with a migration of the isothiocyanate group on the rhamnose moiety. We can also see a fragment at *m*/*z* 169.05 corresponding to the rhamnose structure. Then we performed the MS/MS on the [M + Na]^+^ ion of the complex at *m*/*z* 1306.39 (Figure 12B). It should be noted that unusually high collision energy (95 eV) had to be applied to efficiently fragment the precursor ion. We mainly observed the loss of MOR (−311u) giving the free α-CD at *m*/*z* 995.31 and four successive losses of glucopyranose units (−162u) as currently observed for CDs [32]. However, there was a noticeable loss (−146u) from the precursor ion at *m*/*z* 1306.39 to obtain a fragment at *m*/*z* 1160.33 where accurate mass measurement (1160.3311) allowed its elemental composition determination as being C_44_H_67_O_31_NSNa. This formula corresponds to α-CD associated with *p*-hydroxybenzyl ITC and highlights an unexpected and specific loss (146.0579u) of the rhamnose part (C_6_H_10_O_4_) when MOR is involved in a supramolecular assembly.

**Figure 13:**
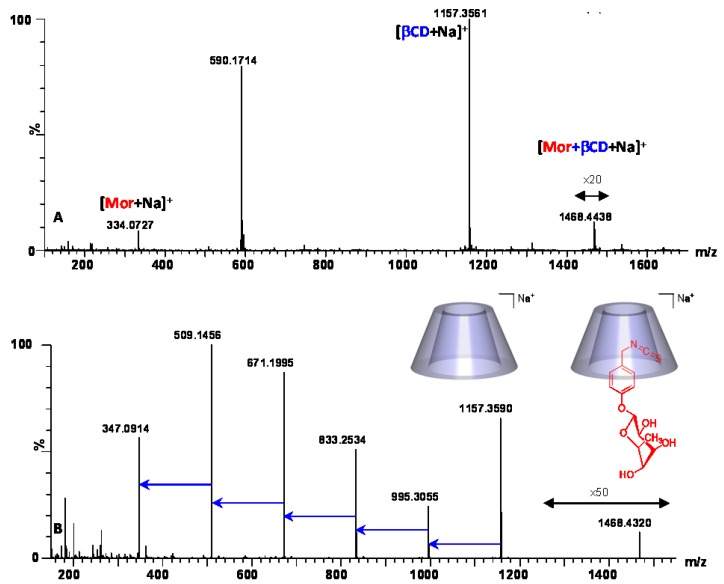
MS (**A**) and MS/MS (**B**) of equimolar mixture of β-CD/MOR in water (0.5 mM) recorded in ESI^+^. The MS/MS spectrum (**B**) was obtained at a collision energy of 95 eV, as applied for α-CD/MOR study (Figure 12B).

We can conclude that this part of MOR is preferentially trapped and stabilized into the α-CD cavity modifying the MOR fragmentation pathway. In order to verify that this protective property is specific to α-CD, we investigated the behavior of β-CD/MOR interaction using similar MS and MS/MS experiments (Figure 13). As for α-CD/MOR mixture (Figure 6A) a sodium adduct at *m*/*z* 1468.44 corresponding to an association with a 1:1 stoichiometry was observed on the MS spectrum (Figure 13A). On the MS/MS spectrum (Figure 13B), we noted that the β-CD/MOR complex ion directly dissociates to the free β-CD at *m*/*z* 1157.35 and its usual losses of glucopyranose units (−162u). The specific loss of the rahmnose part (−146u) previously reported for α-CD/MOR complex (Figure 12B) was not highlihted in the case of β-CD/MOR interaction. This result clearly evidenced that the α-CD/MOR supramolecular self-assembly was the most stable and motivated the use of this more expensive CD for our formulation.

It should be noted that only few similar examples are reported in literature. Twenty years ago, Mele et al. carried out fast-atom bombardment (FAB) mass spectrometric and MS/MS studies of glycoconjugates and their 1:1 association complex with β-CD [33]. Modifications of fragmentation patterns of the guests were observed in presence of β-CD and were attributed to attractive interaction between external surface of β-CD and carbohydrate residues of the guests. More recently, Volmer et al. explored the influence of β-CD on specific peptides by electron capture dissociation (ECD) mass spectrometry studies [34]. Their results showed that the presence of β-CD could reduce the formation of isomerization products during peptide deamidation by formation of inclusion complexes via hydrogen bonding.

Based on the MS/MS results, α-CD can be considered as a “chaperone-like” molecule that assists and modifies the fragmentation pattern of MOR by supramolecular interactions, thus leading to a *p*-hydroxybenzyl ITC/α-CD complex. However, the driving forces seem to be more interaction of *p*-hydroxybenzyl ITC for the cavity of α-CD than the interactions rhamose-cyclodextrin via hydrogen bonds. *p*-hydroxybenzyl ITC is currently obtained by enzymatic hydrolysis of sinalbin, a major glucosinolate found in the seeds of white mustard, *Sinapis alba*. Considering the recognized biological properties of that ITC [35,36] together with its marked instability in aqueous media [37], it would be advisable to further investigate the properties of its CD-inclusion complex with regard to solubility, stability and bioavailability.

In conclusion, the relevance of a structural study, jointly using NMR and mass spectrometry, for an inclusion complex in solution has been established. It was shown that the increase in solubility, stability and bioavailability of MOR in this new formulation was due to the formation of a strong inclusion complex with α-CD.

## 3. Materials and Methods

### 3.1. Materials

α-CD and β-CD were purchased from Wacker Chimie (Lyon, France). For liquid chromatography and mass spectrometry, water, methanol and formic acid (ULC-MS grade) were purchased from Biosolve (Dieuze, France). D_2_O for NMR experiments was purchased from CortecNet (Voisins le Bretonneux, France). GMG was isolated from *M. oleifera* seeds according to previously reported methods [9,20]. The purity was assayed by HPLC analysis of the desulfo-derivative according to the ISO 9167-1 method, yielding about 99% based on peak area value and more than 95% on weight basis due to its marked hygroscopic properties. The enzyme myrosinase was isolated from white mustard (*Sinapis alba* L.) seeds according to a reported method [38]. MOR was produced via myrosinase-catalyzed hydrolysis of GMG, performed in 0.1 M phosphate buffer pH 6.5 at 37 °C. The total conversion of pure GMG into MOR was confirmed by HPLC analysis of the desulfo-derivative [38], which allowed us to monitor the reaction until complete disappearance of GMG in the reaction mixture. Acetonitrile was then added to the mixture until the final concentration was 20% and MOR was purified by reverse-phase chromatography, according to the procedure previously described [9]. MOR was characterized by ^1^H- and ^13^C-NMR and mass spectrometry techniques [3,4].

The complex used for biological evaluations was produced by dissolving in a vial 300 mg of α-CD in 3.0 mL of water to which 103 mg of solid MOR was added. The mixture was then heated to 37 °C and manually stirred until complete solubilization of the compound. The solution was filtered with 0.45 µm filter and freeze-dried.

### 3.2. In Vitro RAW Macrophages Culture Conditions and Drug Treatment

The murine macrophage cell line RAW 264.7 was purchased from Centro substrati cellular, Istituto Zooprofilattico Sperimentale della Lombardia e dell’Emilia, Bologna Italy. Cells were cultured in RPMI-1640 medium (Sigma-Aldrich Co. Ltd., Saint-Louis, MO, USA) containing 10% fetal bovine serum (Sigma-Aldrich Co. Ltd., USA). Cells were grown at 37 °C in a moisturized atmosphere of 5% CO2 and 95% air. Experiments were performed in cells not surpassing 30 passages. For drug treatment, cells were grown to 70–80% confluence followed by 2 h pretreatment with moringin (MOR; 2.5 μM in 1X phosphate buffered saline [PBS]) or α-cyclodextrin (α-CD) conjugated moringin (α-CD/MOR; 2.5 μM in 1X PBS). Then, in order to induce inflammation, the cells were stimulated with lipopolysaccharides from *Escherichia coli* 0111:B4 (LPS) (1 μg/mL; Sigma-Aldrich Co. Ltd., Saint-Louis, MO, USA) for 24 h by adding LPS directly into MOR-treated cell culture medium or α-CD/MOR-treated cell culture medium. Control untreated cells, LPS alone, MOR alone (2.5 μM), α-CD alone (2.5 μM) and α-CD + MOR alone (2.5 μM)-treated cells were also included as controls. After LPS stimulation, the cells were fixed for eosin and hematoxylin staining. Additionally, in order to evaluate the stability of the compounds, MOR and α-CD + MOR solutions were stored at room temperature for 1 week and a similar set of experiments was performed to evaluate the cytoprotective effect against LPS-stimulated macrophages.

### 3.3. Eosin and Hematoxylin (E&H) Staining

Cells on coverslips (10 mm; Thermo Scientific, Karlsruhe Germany) were fixed with 4% paraformaldehyde (Santa Cruz, Dallas, TX, USA) at room temperature for 15 min followed by 1X PBS (pH 7.5) washes. Morphological changes in the cells were assessed by eosin and hematoxylin staining (E&H). Microscopy was performed using light microscopy (LEICA DM 2000 combined with LEICA ICC50 HD camera, LEICA Microsystems, Wetzlar, Germany). All images are representative of three independent experiments.

### 3.4. Nuclear Magnetic Resonance (NMR) Studies

All NMR experiments were performed at 600.17 MHz using a Bruker Avance III spectrometer (Bruker Biospin, Wissembourg, France) equipped with a Z-gradient unit for pulsed-field gradient spectroscopy and with a 5 mm TXI probe. Calibration was performed using the signal of the residual protons of the solvent (HOD) as a reference. Measurements were performed at 300 K with careful temperature regulation. The length of the 90° pulse was approximately 7 µs. ^1^D-NMR data spectra were collected using 16 K data points. 2D experiments were run using 1 K data points and 512 time increments. The phase sensitive (TTPI) sequence was used and processing resulted in a 1 K × 1 K (real-real) matrix. Details of the experimental conditions are given in the figure captions. For diffusion coefficient measurements, 2D ^1^H DOSY NMR experiments were carried out using the Bruker sequence ledbpgp2s. Gradient calibration of the probe was done using the water signal from an H_2_O/D_2_O (90/10) mixture; the gradient value G = 4.9 G/mm was obtained for a water diffusion value D at 2.3 × 10^−9^ m^2^/s at 25 °C. The strength of the pulsed-field gradient was linearly increased from 2 to 95% in 16 steps. The diffusion time (Δ) and the gradient duration (δ/2) were set at 80 ms and 1.5 ms, respectively. The longitudinal eddy current delay and the spoil gradient delay were fixed at 5 and 0.2 ms, respectively. Spectral data were processed via the dosy2d module from TOPSPIN software (version 3.2, Bruker Biospin, Wissembourg, France). Fourier transform was applied in the F2 dimension for every FID obtained from each programmed gradient value and a baseline correction was applied on every one-dimensional spectrum. An automatic search of the intensity decay for each peak was carried out to obtain the decay time proportional to D. Diffusion coefficient values obtained were expressed according to the F1 dimension.

### 3.5. Solubility Studies

Phase solubility studies were carried out according to the modified Higuchi and Connors method. Excess amount of MOR were added (20 mg) to a 0.6 mL D_2_O solution containing different α-CD concentrations (from 10 to 50 mM). The suspensions were maintained at 300 K for 24 h, filtered through 0.2 µm cellulose filters from Grace (Columbia, MD, USA) and analysed by a ^1^H-NMR quantitative experiment called ERETIC [24]. First, a ^1^H-NMR experiment using zg pulse program (D1 5 s, AQ 4.54 s, TD 64K, NS 8) with a Bruker reference standard triphenylphosphate (TPP) (48.5 mM in aceton-d6)was performed. This reference spectrum obtained for TPP with a known and accurate concentration enabled to assign the integrated signals to 15 protons and was used to externally calibrate and quantify MOR at saturation in the absence and in the presence of α-CD in D_2_O. The described phase solubility diagram was obtained by using integration of H1’ signal of MOR to determine its concentration.

### 3.6. Determination of the Stoichiometry

Job plot procedure was applied: ^1^H-NMR spectra of 11 samples of mixture of α-CD and MOR were recorded in D_2_O. The sum of both species was kept constant at 10 mM; the molar ratio *r* of each component was varied from zero to one. The observation of any chemical shift of the host or guest varying in a linear fashion with the concentration of bound species was obtained and affords the corresponding job plots as described elsewhere [26].

### 3.7. Determination of the Associate Constant Value

The concentration of MOR was kept constant at 0.2 mM whereas the concentration of α-CD was varied from 5 to 40 mM. Measurements of MOR chemical shift were performed at 300 K with careful temperature regulation [30]

### 3.8. Mass Spectrometry (MS) Studies

Automated flow injections were performed using an ACQUITY UPLC H-Class system (Waters, Manchester, UK) coupled to a SYNAPT G2-Si-Q-TOF hybrid quadrupole time-of-flight instrument (Waters, Manchester, UK) equipped with an electrospray ionization (ESI) source (Z-spray) and an additional sprayer for the reference compound (Lock Spray). The mobile phase was composed of water and the flow rate was set to 0.4 mL/min. Water solutions of MOR, α-CD, β-CD and MH alone (0.5 mM) and equimolar mixtures of α-CD/MOR, β-CD/MOR, MH/MOR (0.5 mM) were analyzed by ESI-HRMS and MS/MS in the positive ionization mode. One microliter of each sample was injected, and the acquisition method run time was 2 min. The electrospray ionization used a capillary voltage of 3 kV for positive mode and the following specific conditions: sampling cone voltage, 50 V; source offset, 20 V; source temperature, 120 °C; desolvation gas temperature, 250 °C; desolvation gas flow, 600 L/h, and cone gas flow, 50 L/h. Nitrogen (>99.5%) was employed as desolvation and cone gas. Mass calibration was performed using a sodium formate solution and Leu-enkephalin (*m*/*z* 556.2771) was used as the lock mass solution for accurate mass measurements. The scan range was *m*/*z* 50–2000 at 0.2 s/scan. The time of flight (TOF) was operated in the resolution mode, providing an average resolving power of 25,000 (FWHM). The HMRS spectra were recorded in the centroid mode. For MS/MS experiments, argon was used as collision gas and the collision energy was optimized for each [M + Na]^+^ precursor ion. For IM-MS experiments, separation of ions in gas phase was achieved in the travelling wave ion mobility cell using nitrogen as drift gas and the following wave velocity parameters: (WV) = 600 m/s, wave height (WH) = 40 V, to obtain mobility traces of each sample. Data acquisition was performed with MassLynx software (V4.1, Waters).

### 3.9. Modelization Studies

Molecular dynamics were performed using the OPLS force field, with an implicit simulation of water (Generalized Born model, dielectric permittivity equal to 78), as implemented in the Abalone software. The CD host was based on a non-distorted α-CD structure with C6 symmetry, while MOR was constructed by means of the tools integrated in the software. MOR was then manually introduced within the α-CD cavity, with a careful attention devoted to the observed steric complementarity. An initial optimization step of the inclusion compound was realized (steepest descent algorithm), before launching molecular dynamics simulations (NPT ensemble at constant pressure of 1 atm and temperature of 298 K, integration step 1 fs, equilibration time 1 ns, simulation time 10 ns). All generated structures were used to calculate the interaction energy (sum of the van der Waals and electrostatic interactions between the two partners within the complex) and the complexation energy (difference between the total energies obtained for the complex and for the sum of the isolated species). This procedure was realized in triplicate, in order to calculate mean values of interaction and complexation energies.
